# Preoperative vitamin D level is significantly associated with hypocalcemia after total thyroidectomy

**DOI:** 10.1186/s12891-022-05977-4

**Published:** 2022-12-22

**Authors:** Yantao Qi, Jixin Chai, Liuyang Zhang, Yong Chen

**Affiliations:** Department of Thyroid Surgery, The Affiliated Hospital of Chengde Medical University, No.36, Nanyingzi Street, 067000 Chengde, Hebei Province China

**Keywords:** Vitamin D deficiency, Hypocalcaemia, Total thyroidectomy, Risk factor

## Abstract

**Background:**

To evaluate the association of preoperative vitamin D levels with postoperative hypocalcaemia after total thyroidectomy.

**Methods:**

The medical records of patients who underwent total thyroidectomy between May 2020 and January 2022 and who had a documented preoperative serum 25-hydroxyvitamin D (25-OHD) concentration were retrospectively reviewed. Vitamin D levels were categorized into four groups: <10 ng/mL (severe vitamin D deficiency), 10–20 ng/mL (vitamin D deficiency), 20–30 ng/mL (vitamin D insufficiency), and > 30 ng/mL (vitamin D sufficiency). Multivariate logistic regression was performed to analyse the association of vitamin D levels with the risk of hypocalcaemia after controlling for potential confounding factors.

**Results:**

A total of 196 patients were included in this study. Of these, 47 (24.0%) had preoperative 25-OHD < 10 ng/mL, 62 (31.6%) had 25-OHD of 10–20 ng/mL, 51 (26.0%) had 25-OHD of 20–30 ng/mL and the remaining 36 (18.4%) had 25-OHD > 30 ng/mL. The incidence of postoperative hypocalcemia was highest in the group of patients with severe vitamin D deficiency (42.6% and 23.4% for postoperative laboratory and symptomatic hypocalcaemia, respectively), followed by the group with vitamin D deficiency (29.0% and 16.1%), the group with vitamin D insufficiency (19.6% and 5.9%) and the group with vitamin D sufficiency (5.6% and 2.8%). Multivariate logistic regression indicated that the odds of postoperative laboratory hypocalcaemia for patients with severe vitamin D deficiency and vitamin D deficiency were 13.20 times (95% CI: 2.69–64.79, *P* < 0.01) and 6.32 times (95% CI: 1.32–30.28, *P* = 0.02) greater than for those with vitamin D sufficiency, respectively; while the odds of symptomatic hypocalcaemia for patients with severe vitamin D deficiency was 10.18 times (95% CI: 1.14–90.86, *P* = 0.04) greater than for those with vitamin D sufficiency.

**Conclusion:**

Preoperative vitamin D deficiency (< 20 ng/mL), especially severe vitamin D deficiency (< 10 ng/mL), is an independent predictive factor of postoperative hypocalcaemia after total thyroidectomy.

## Background

Total thyroidectomy is the recommended procedure for a large proportion of thyroid malignancies and benign thyroid pathologies such as Graves’ disease and multinodular goiter. Postoperative hypocalcaemia is the most common complication. As shown in a retrospective cross-sectional study, approximately 19.1% of 126,766 patients who underwent total thyroidectomy developed postoperative hypocalcaemia within 30 days of surgery [1]. Hypocalcaemiacan be asymptomatic, but the condition is sometimes severe and can lead to acute life-threatening conditions, such as tetany, laryngospasm, confusion, seizures, arrhythmias, and heart failure [2]. Although most patients with hypocalcaemia recover within a few months, up to 13% may be permanently affected [3].

Despite an increasing number of studies investigating predictors of postoperative hypocalcaemia, there have been conflicting results regarding the impact of preoperative vitamin D deficiency. A meta-analysis of risk factors for hypocalcaemia after total thyroidectomy pooling the results of eight studies indicated that the incidence of hypocalcaemia was significantly increased in patients with vitamin D deficiency [4]. In contrast, several recently published studies concluded that preoperative vitamin D levels could not predict the risk of postoperative hypocalcaemia [5–7]. Some of the reasons for these discordant results could be differences in the criteria used to diagnose hypocalcaemia, diverse cut points for 25-OHD, and study group heterogeneity.

The present study aims to evaluate the potential for using preoperative serum vitamin D concentrations to predict postoperative hypocalcaemia after total thyroidectomy in a homogenous group of patients.

## Methods

### Patients

This study comprised a retrospective review of all thyroidectomy operations performed at our hospital between May 2020 and January 2022. The inclusion criteria were the performance of total thyroidectomy on adult patients (≥ 18 years) and a documented 25-OHD concentration obtained within one week of the date of surgery. Exclusion criteria were preoperative hypercalcaemia, previous thyroid surgery or irradiation, coexisting parathyroid disease, usage of a drug that interfered with calcium homeostasis, and renal insufficiency.

## Data collection and outcomes

Preoperative variables including patient demographics, and preoperative laboratory measurements for 25-OHD, serum calcium, phosphate, parathyroid hormone (PTH), and albumin concentrations were measured within one week before surgery in all patients. Postoperative calcium levels were generally measured at 6 AM and 5 PM on the first postoperative day and 6 AM on the second postoperative day. PTH was measured at the same time as the first calcium measurement on postoperative Day 1.

Patients included in the analysis were divided into four groups according to vitamin D levels: severe deficiency (25-OHD < 10 ng/mL), deficiency (25-OHD of 10–20 ng/mL), insufficiency (25-OHD of 20–30 ng/mL) or sufficiency (25-OHD > 30 ng/mL).

The primary outcomes evaluated were the occurrence of postoperative laboratory hypocalcaemia and symptomatic hypocalcaemia. Laboratory hypocalcaemia was defined as the occurrence of any single adjusted serum calcium concentration less than 8 mg/dl. Serum calcium concentration was adjusted for serum albumin: adjusted calcium = 0.8*(4.0-serum albumin) + serum calcium. Symptomatic hypocalcaemia was defined as subjective or objective symptoms such as tingling, numbness, or carpopedal spasm with laboratory hypocalcaemia. When symptomatic hypocalcaemia developed during a hospital stay, calcium and vitamin D were administered orally. If the symptoms persisted, calcium gluconate was administered intravenously.

### Statistical analysis

Continuous variables with normal distribution are presented as means ± SD and compared with the use of One-way Analysis of Variance (ANOVA), followed by *post-hoc* analysis. All categorical variables were summarised and expressed as proportions and compared with the use of the chi-square test with normal approximation or Fisher’s exact test, as appropriate. Multivariate logistic regression was performed to analyse the association of vitamin D levels with the odds of hypocalcaemia after controlling for potential confounding factors. All tests were 2-sided and a *P* value of less than 0.05 was considered significant.

All statistical analyses were performed with the SPSS statistical software program package (SPSS version 20.0 for Windows, Armonk, NY: IBM Corp.).

## Results

A total of 196 patients were included in this study. Of these, 47 (24.0%) had preoperative severe vitamin D deficiency, 62 (31.6%) had vitamin D deficiency, 51 (26.0%) had vitamin D insufficiency and the remaining 36 (18.4%) had vitamin D sufficiency. The baseline demographics and clinical profiles for the four groups are presented in Table 1. Differences in age, sex, BMI, residence, season of surgery, and preoperative serum calcium, albumin, and PTH were not statistically significant among the four groups (all *P* > 0.05).

**Table 1:** Patients’ baseline demographics and clinical characteristics

	Vitamin D category				*P* value
	**Severe deficiency ** **(25-OHD < 10 ng/mL)**	**Deficiency** **(25-OHD 10–20 ng/mL)**	**Insufficiency ** **(25-OHD 20–30 ng/mL)**	**Sufficiency ** **(25-OHD > 30 ng/mL)**	
N	47 (24.0%)	62 (31.6%)	51 (26.0%)	36 (18.4%)	
Age (yrs)	46.4 ± 5.1	47.0 ± 5.2	477.1 ± 5.6	46.3 ± 5.1	0.85
Gender					
Female	35 (74.5%)	46 (74.2%)	62 (71.3%)	62 (71.3%)	0.89
Male	12 (25.5%)	16 (25.8%)	25 (28.7%)	25 (28.7%)	
BMI (kg/m^2^)	23.8 ± 3.3	24.2 ± 3.4	24.8 ± 3.4	23.4 ± 44.0	0.28
Residence					
Urban	32 (68.1%)	46 (74.2%)	58 (66.7%)	58 (66.7%)	0.60
Rural	15 (31.9%)	16 (25.8%)	29 (33.3%)	29 (33.3%)	
Season of surgery					0.29
Spring	8 (17.0%)	12 (19.4%)	11 (21.6%)	6 (16.7%)	
Summer	17 (36.2%)	21 (33.9%)	10 (19.6%)	6 (16.7%)	
Autumn	15 (31.9%)	14 (22.6%)	13 (25.5%)	13 (36.1%)	
Winter	7 (14.9%)	15 (24.2%)	17 (33.3%)	11 (30.6%)	
Preoperative 25-OHD (ng/ml)	7.0 ± 1.8	16.5 ± 2.3	25.7 ± 2.8	33.2 ± 22.0	< 0.01
Preoperative serum calcium (mg/dl)	8.9 ± 0.3	8.9 ± 0.3	8.9 ± 0.2	8.8 ± 0.3	0.16
Preoperative serum albumin (g/dL)	3.2 ± 0.7	3.2 ± 0.8	3.2 ± 0.7	3.2 ± 0.6	0.97
Preoperative PTH (pg/ml)	54.4 ± 14.9	51.7 ± 14.4	51.0 ± 16.1	48.5 ± 14.1	0.36

**Fig. 1:**
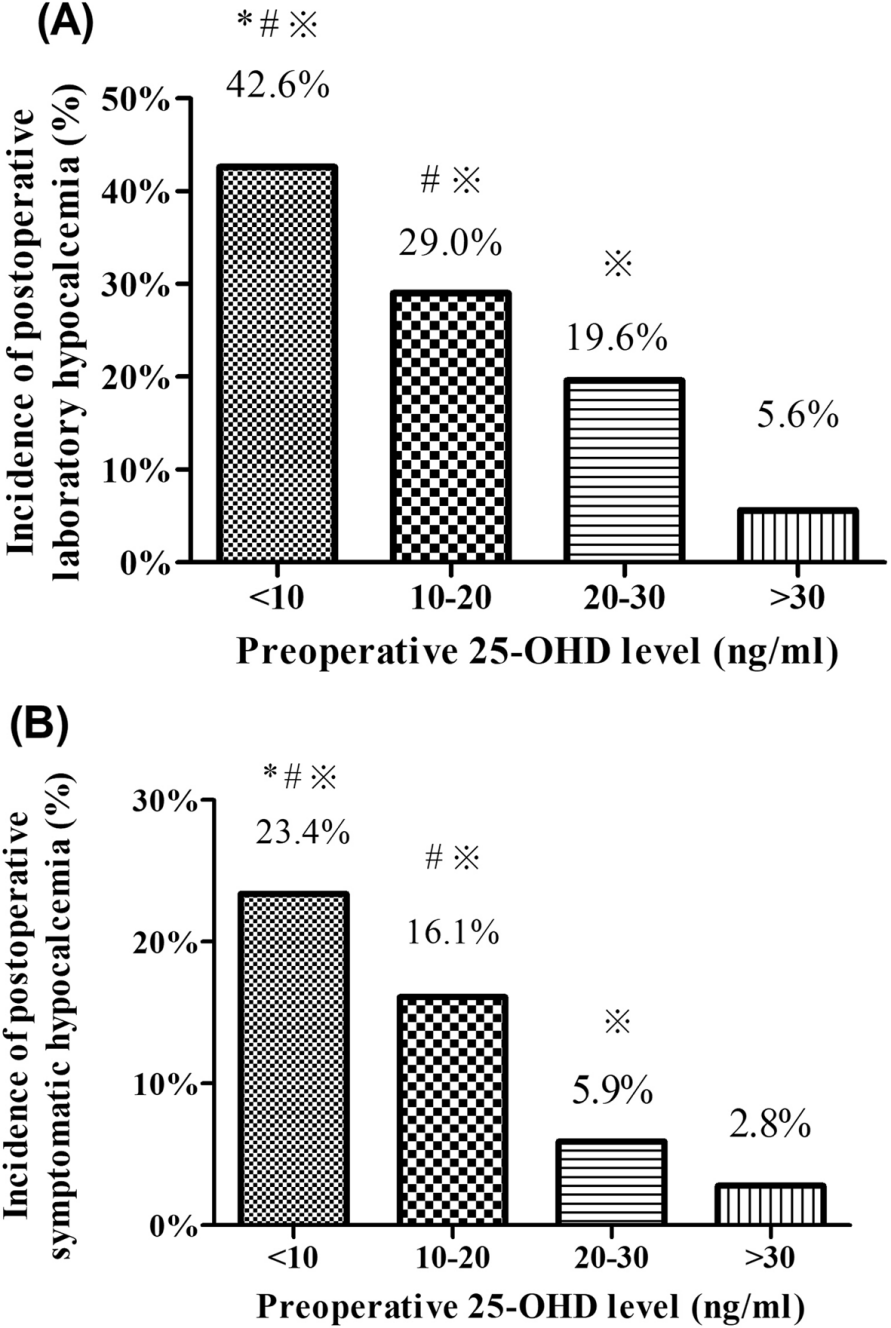
Incidences of postoperative laboratory and symptomatic hypocalcemia according to preoperative 25-OHD levels. ^*^ *P* < 0.05 compared with 25-OHD 10–20 ng/mL; ^#^ *P* < 0.05 compared with 25-OHD 20–30 ng/mL; ^※^ *P* < 0.05 compared with 25-OHD > 30 ng/mL

Postoperatively, 50 (25.5%) patients developed hypocalcaemia, and 25 (12.8%) had symptoms of hypocalcaemia. As shown in Fig. 1, the incidence of postoperative hypocalcemia was highest in the group of patients with severe vitamin D deficiency (42.6% and 23.4% for postoperative laboratory and symptomatic hypocalcaemia, respectively), followed by the group with vitamin D deficiency (29.0% and 16.1%), the group with vitamin D insufficiency (19.6% and 5.9%) and the group with vitamin D sufficiency (5.6% and 2.8%).

**Fig. 2:**
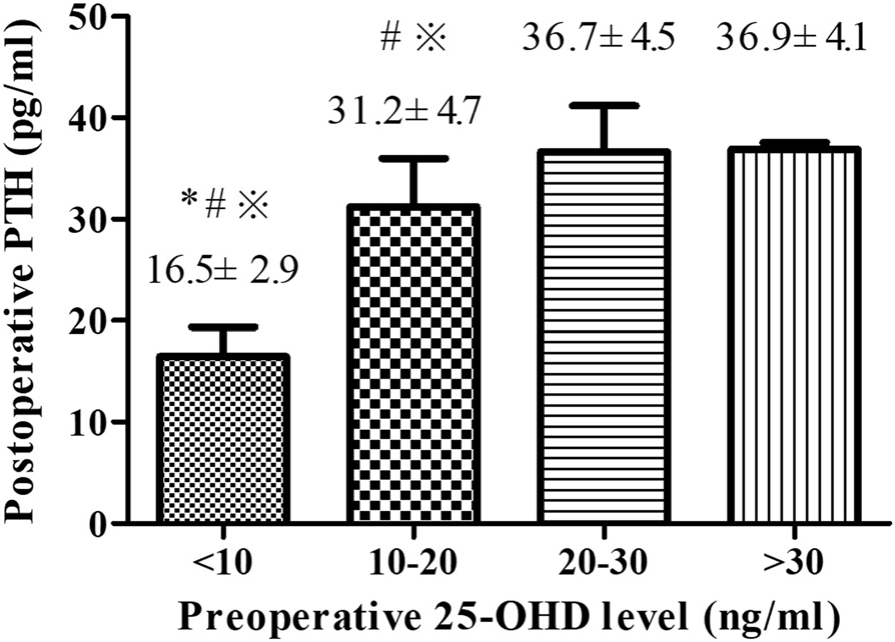
Postoperative parathyroid hormone (PTH) levels according to preoperative 25-OHD levels. ^*^ *P* < 0.05 compared with 25-OHD 10–20 ng/mL; ^#^ *P* < 0.05 compared with 25-OHD 20–30 ng/mL; ^※^ *P* < 0.05 compared with 25-OHD > 30 ng/mL

Mean postoperative PTH levels are reported in Fig. 2 according to the four categories of preoperative serum 25-OHD levels. Postoperative PTH levels in patients with severe vitamin D deficiency (16.5 ± 2.9 pg/mL) were significantly lower than those in patients with vitamin D deficiency (31.2 ± 4.7 pg/mL), vitamin D insufficiency (36.7 ± 4.5 pg/mL) and vitamin D sufficiency (36.9 ± 4.1 pg/mL).

Multivariate logistic regression (Table 2) revealed that after controlling for potential confounding factors including age, sex, BMI, residence, and preoperative serum calcium, albumin, and PTH, the odds of postoperative laboratory hypocalcaemia for patients with severe vitamin D deficiency and vitamin D deficiency were 13.20 times (95% CI: 2.69–64.79, *P* < 0.01) and 6.32 times (95% CI: 1.32–30.28, *P* = 0.02) greater than for those with vitamin D sufficiency, respectively; while the odds of symptomatic hypocalcaemia for patients with severe vitamin D deficiency was 10.18 times (95% CI: 1.14–90.86, *P* = 0.04) greater than for those with vitamin D sufficiency.

**Table 2:** Multivariate logistic regression analysis to assess the risk of postoperative laboratory and symptomatic hypocalcemia

	Laboratory hypocalcemia				Symptomatic hypocalcemia		
	**RR**	**95% CI**	***P*** **value**		**RR**	**95% CI**	***P*** **value**
Vitamin D groups							
25-OHD > 30 ng/mL	Reference				Reference		
25-OHD 20–30 ng/mL	3.78	0.74–19.19	0.11		1.99	0.18–21.67	0.57
25-OHD10-20 ng/mL	6.32	1.32–30.28	0.02		7.60	0.86–66.88	0.07
25-OHD ≤ 10 ng/mL	13.20	2.69–64.79	< 0.01		10.18	1.14–90.86	0.04
Age	1.06	0.99–1.14	0.10		1.07	0.98–1.18	0.15
Gender							
Male	Reference						
Female	0.95	0.43–2.09	0.90		0.90	0.31–2.65	0.85
BMI	1.08	0.97–1.20	0.17		1.09	0.95–1.26	0.21
Place of residence							
Urban	Reference						
Rural	0.78	0.35–1.75	0.55		1.68	0.62–4.56	0.31
Season of surgery							
Spring	Reference				Reference		
Summer	0.97	0.33–2.83	0.95		4.56	0.86–24.54	0.08
Autumn	1.48	0.51–4.27	0.47		5.33	0.99–28.59	0.06
Winter	0.90	0.30–2.77	0.86		1.40	0.20–9.61	0.74
Preoperative serum calcium	3.73	0.97–14.36	0.06		0.38	0.07–2.13	0.27
Preoperative serum albumin	0.75	0.44–1.26	0.27		0.70	0.36–1.34	0.28
Preoperative PTH	1.00	0.97–1.02	0.70		1.01	0.98–1.04	0.56

## Discussion

Hypocalcaemia is a well-known postoperative complication of thyroidectomy. Consistent with the incidence of hypocalcaemia ranging from 19 to 38% reported by a meta-analysis and several recent publications [1, 8, 9], laboratory hypocalcaemia was found in 23.8% of patients in this retrospective study. Although hypocalcaemia is often asymptomatic, some clinical symptoms including tingling, numbness, or carpopedal spasm can also be seen, which can limit the routine daily activities of such patients. Symptomatic hypocalcemia was found in 12.8% of patients, which is also consistent with previous studies [9]. In addition, the present study showed that preoperative vitamin D deficiency, especially severe vitamin D deficiency, is associated with an increased risk of postoperative hypocalcaemia.

Vitamin D plays a vital role in the regulation of PTH and calcium [10]. It increases serum calcium by directly increasing calcium absorption from the intestine and bone resorption, and regulating the secretion of PTH from the parathyroid glands [11, 12]. Thus, preoperative vitamin D may exert a profound effect on the perioperative kinetics of calcium and PTH postthyroidectomy [13]. When parathyroid function is impaired, sufficient vitamin D can promote the absorption of intestinal calcium and maintain calcium homeostasis. However, preoperative vitamin D deficiency damaged the regulatory mechanism in patients undergoing total thyroidectomy [14, 15], which increased the incidence of postoperative hypocalcaemia and led to hypoparathyroidism due to parathyroid ischaemia/injury or inadvertent resection. Several studies [16, 17] reported that as the vitamin D level decreased, the calcium level decreased statistically significantly while PTH increased. However, significant associations were not found in the current study. This may be due to the limited sample size in our study as we did not aim to explore the association of preoperative vitamin D levels with calcium and PTH levels.

In previous studies, many different cutoff levels for vitamin D have been explored to predict the risk of postoperative hypocalcaemia. We used three widely accepted cutoff levels of 10, 20, and 30 ng/mL and found that the incidence of postoperative hypocalcaemia was significantly higher for patients with 25-OHD < 10 ng/mL than for those with 25-OHD > 30 ng/mL. Our results are consistent with many previous studies. Al-Khatib and colleagues performed multivariate analysis on 213 patients undergoing total and completion thyroidectomy and showed that severe vitamin D deficiency, defined as serum 25-OHD < 10 ng/ml, was an independent predictor of postoperative hypocalcaemia [18]. A prospective study conducted by Kirkby-Bott et al. reported a dose-dependent relationship between vitamin D level and the risk of hypocalcaemia as hypocalcaemia in patients with vitamin D levels < 10 ng/ml was significantly more likely than hypocalcaemia in patients with vitamin D levels > 20 ng/ml (32% vs. 13%, *P* < 0.025) [14]. Another prospective study conducted by Daglar et al. indicated that the patients who had < 10 ng/mL vitamin D levels (severe deficiency) developed significantly more biochemical and clinical hypocalcemia than the patients with serum vitamin D levels higher than 10 ng/mL (*P* = 0.030 and *P* < 0.001, respectively) [9]. However, some studies have reported diverse results. Two studies conducted by Griffin et al. [8] and Lang et al [12] found no correlation between vitamin D levels and the risk of postoperative hypocalcaemia using vitamin D cutoffs of both 10 and 20 ng/mL [8]. The reasons for these conflicting results could be related to differences in the populations as well as patients’ individual characteristics.

As patients with hypocalcaemia may require longer hospitalization, more biochemical studies, extended pharmacological treatments, and additional medical resources, hypocalcaemia has become a burden for the health care system. Thus, some authors have recommended routine supplementation with calcium or vitamin D. A systematic review indicated that 7 out of the 9 trials included reported statistically significantly reduced rates of postoperative laboratory hypocalcaemia (absolute risk reduction, 13–59%) and symptomatic hypocalcaemia (absolute reduction, 11–40%) following preoperative supplementation [19]. In several guidelines, for patients with a laboratory confirmed vitamin D deficiency (i.e., 25-OHD < 20 ng/ml), an age- and body weight- dependent therapeutic dosage was recommended to be used for 1–3 months; the dosage should be as follows (with ranges dependent on body weight): for neonates 1,000 IU/day; for infants 2,000–3,000 IU/day; for children and adolescents aged 1–18 years 3,000–5,000 IU/day; for adults and the elderly 7,000–10,000 IU/day or 50,000 IU/week [20]. Previous studies have shown that treatment with vitamin D_3_ (cholecalciferol) takes three to five days to raise serum 25-OHD [21, 22]. In contrast, a single oral dose of calcifediol can generate the needed 25-OHD concentration within four hours [22, 23]. Considering that both D_3_ and 25-OHD enter immune cells to generate calcitriol, using the combination of D_3_ (medium-term) and calcifediol (immediate) is cost-effective and leads to the best clinical outcome [22].

Limitations of this study include its retrospective design and findings. Some preoperative and postoperative parameters might be missing, such as data on hypocalcaemia symptoms. In addition, the risk of permanent hypocalcaemia cannot be evaluated due to the lack of long-term follow-up of serum calcaemic levels.

In conclusion, preoperative vitamin D deficiency (25-OHD < 20 ng/mL), especially severe vitamin D deficiency (25-OHD < 10 ng/mL), is an independent predictive factor of postoperative hypocalcaemia after total thyroidectomy. Routine supplementation of vitamin D using the combination of D_3_ (medium-term) and calcifediol (immediate) with an age- and body weight- dependent therapeutic dosage is recommended for patients with vitamin D deficiency, especially severe vitamin D deficiency, thereby reducing postoperative hypocalcaemia.

## Data Availability

The datasets used and/or analyzedanalyzed during the current study are available from the corresponding author upon reasonable request.
